# Septic Pelvic Thrombophlebitis Status Post-Laparoscopic Hysterectomy: A Case Report Investigating a Rare Yet Perilous Adverse Outcome

**DOI:** 10.7759/cureus.75561

**Published:** 2024-12-11

**Authors:** Maciek Jurecki, Michael Fathalla, Andrea Jeffress

**Affiliations:** 1 Family Medicine, Saint Vincent Hospital, Erie, USA; 2 Obstetrics and Gynecology, Lake Erie College of Osteopathic Medicine, Erie, USA; 3 Obstetrics and Gynecology, Saint Vincent Hospital, Erie, USA

**Keywords:** bipolar electrocautery, gynecologic surgical procedures, hysterectomy, laparoscopy, postoperative fever, septic pelvic thrombophlebitis

## Abstract

Septic pelvic thrombophlebitis is defined as an endovascular thrombus of infectious etiology. It is frequently diagnosed only after excluding other more common pathologies. A high level of suspicion should be maintained in the context of a fever refractory to broad-spectrum antibiotics that improves after initiation of systemic anticoagulation. The risk of thromboembolic events following minimally invasive surgery is minute; however, it requires increased awareness, as prompt management is critical for decreasing morbidity and mortality.

We discuss a case of septic thrombophlebitis in a 42-year-old female who was postoperative day six status post elective total laparoscopic hysterectomy, bilateral salpingectomy, and right ovarian cystectomy due to abnormal uterine bleeding. The case was complicated by a postoperatively diagnosed small bowel injury that necessitated resection. The patient continued to exhibit a fever of unknown origin despite the administration of broad-spectrum antibiotics; therefore, CT imaging with IV contrast was obtained. The imaging raised concerns about septic thrombophlebitis, and the symptoms were resolved with the administration of a 16-hour infusion of intravenous heparin.

Despite its rarity, septic pelvic thrombophlebitis should be considered in patients with relevant risk factors and persistent fever refractory to antibiotics status post-laparoscopy. This report highlights the importance of awareness of SPT and encourages further research on thromboembolic events in laparoscopic surgeries.

## Introduction

Septic thrombophlebitis is defined as an endovascular thrombus in the setting of bacterial or fungal infection and is most commonly seen within venous structures [1]. Ovarian vein thrombosis (OVT) is an infrequent yet potentially severe postoperative complication, often associated with gynecologic procedures such as cesarean sections, oophorectomies, and hysterectomies [2]. OVT is most frequently observed in the postpartum period [2]. The clinical presentation can be elusive and nonspecific, with fever and abdominal pain being the most prevalent symptoms, thus making the diagnosis a challenge. However, suspicion should be high as a delay in diagnosis can lead to complications such as ovarian abscess, ovarian infarction, septic thrombophlebitis, extension into the inferior vena cava (IVC), pulmonary embolization, and uterine necrosis [3]. Diagnostic modalities, such as CT scans, MRI, and ultrasonography, can aid in the identification of this condition. The standard treatment protocol typically involves the administration of antibiotics and anticoagulants [1,2,4,5].

The purpose of this report is to shed light on a rare case of OVT in a 42-year-old female who underwent a total laparoscopic hysterectomy, bilateral salpingectomy, and right ovarian cystectomy. We postulate that the surgical technique employed, specifically the thermal damage induced by bipolar electrocautery, should be considered as a potential contributing factor to the development of OVT. When electrocautery is used, temperatures rapidly rise above 100°C, leading to cellular vaporization and explosion. In vascular structures, this thermal damage can lead to endothelial injury, therefore disrupting the integrity of vessel walls. This immense heat can cause thermal injury to surrounding vasculature, denaturation of proteins, and even thrombosis.

## Case presentation

**Figure 1:**
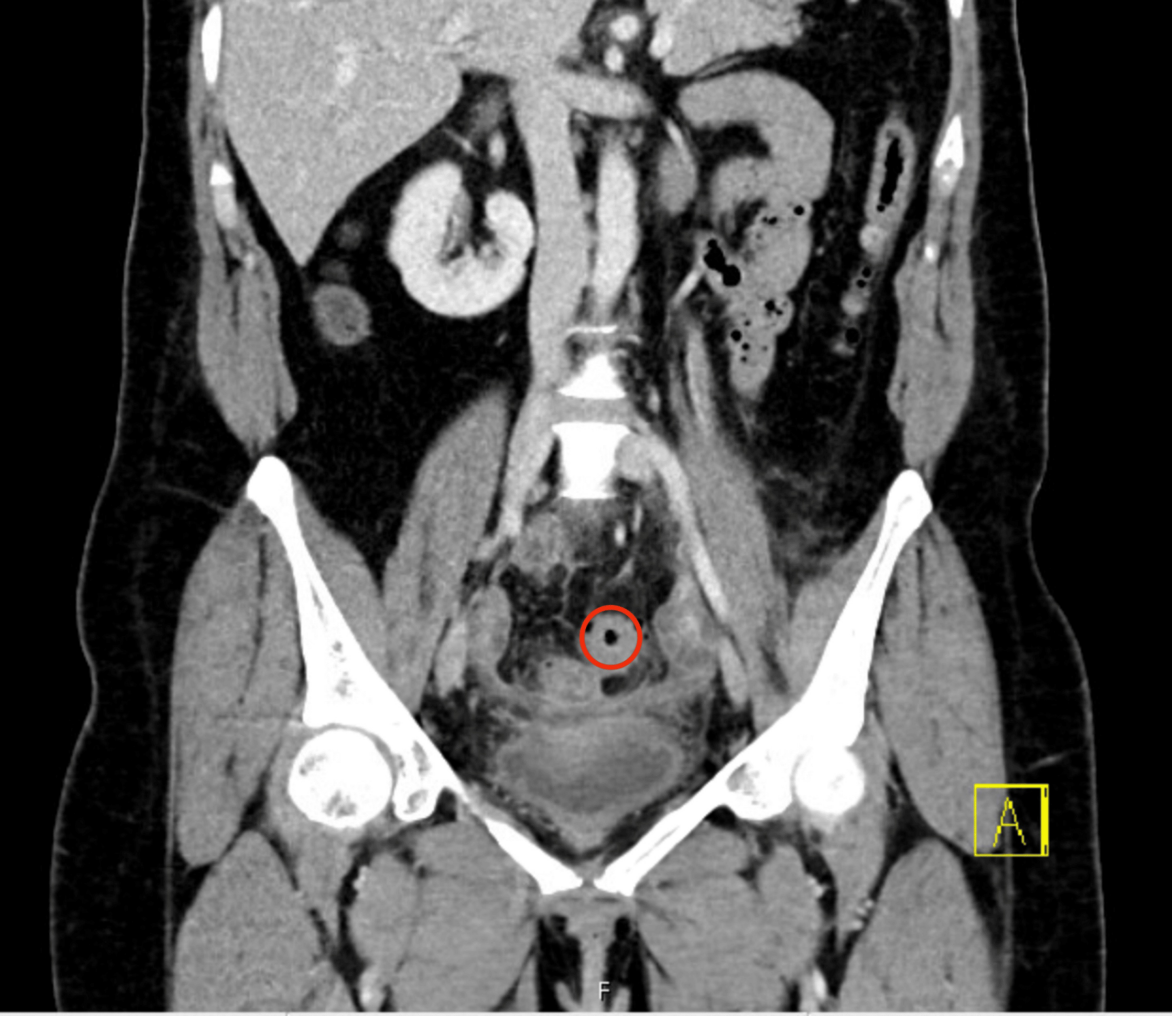
Transverse CT of the abdomen and pelvis demonstrating extraluminal air-fluid collection within the left pelvis

**Figure 2:**
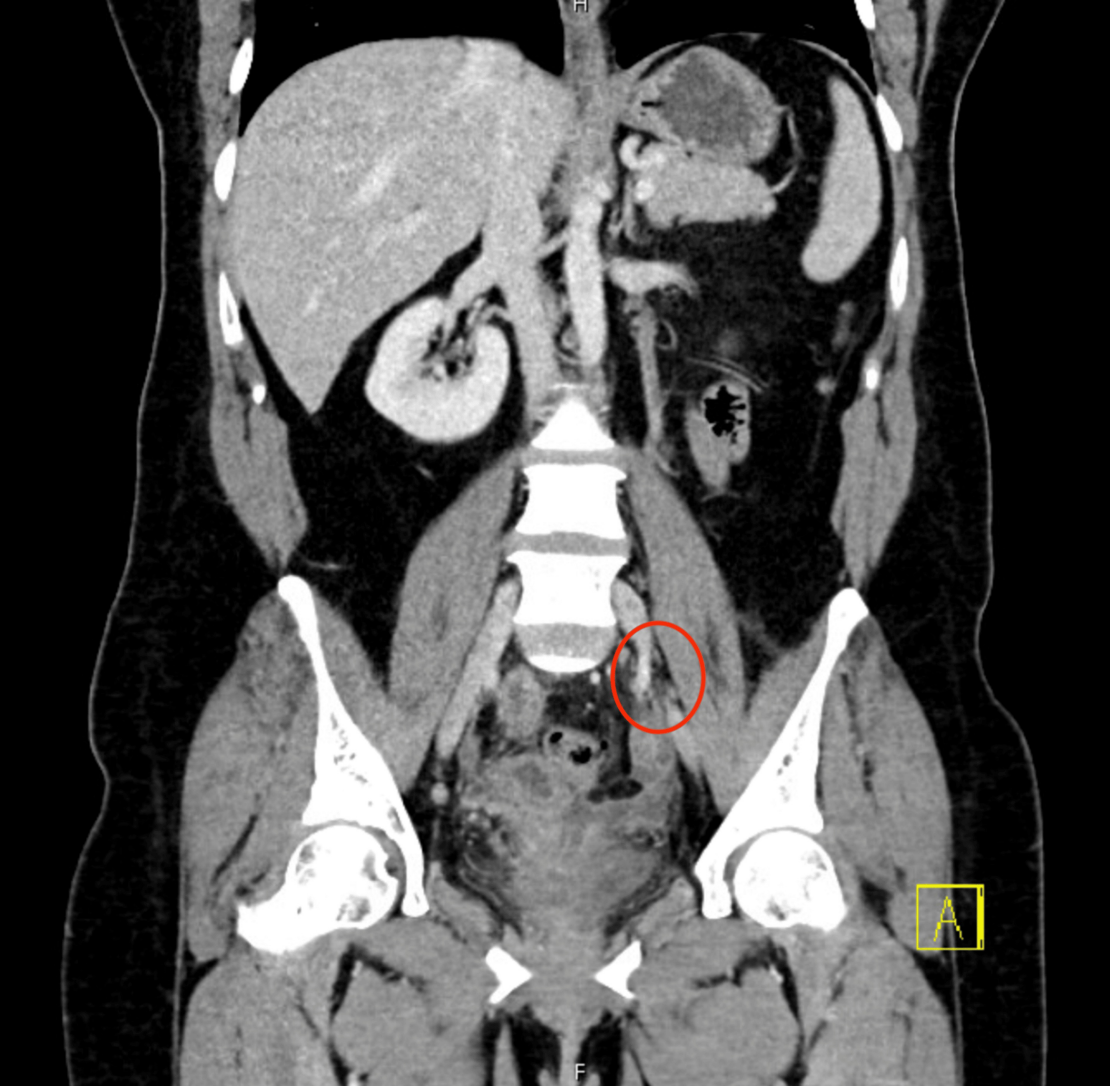
Coronal CT of the abdomen and pelvis demonstrating left gonadal vein filling defect

**Figure 3:**
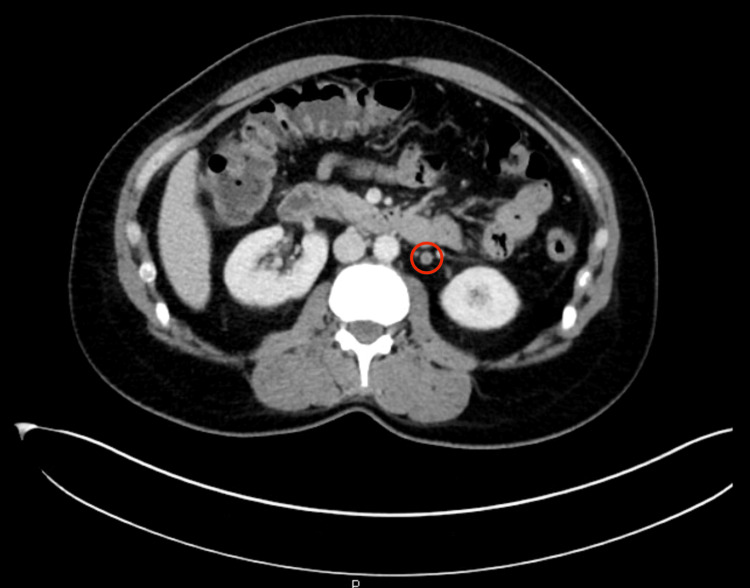
Transverse CT of the abdomen and pelvis demonstrating left gonadal vein filling defect

A 42-year-old female presented to the emergency department due to a two-day history of increasingly worsening lower abdominal pain and night sweats with rigors. Past medical history was negative for hypercoagulability and immunocompromised state. The patient was postoperative day 6 status post elective total laparoscopic hysterectomy and bilateral salpingectomy due to menorrhagia refractory to endometrial ablation. The patient had also undergone a right ovarian cystectomy due to an incidental right ovarian cyst. Preoperative risk assessment for venous thromboembolism (VTE) yielded a Caprini score of 0.7%; hence, preoperative anticoagulation was not administered. Based on this score, postoperative VTE chemoprophylaxis was not indicated; therefore, sequential compression devices were utilized. Adequate hydration status was maintained with a continuous infusion of maintenance fluids, and early ambulation was encouraged. Preoperative antibiotics consisted of cefazolin and metronidazole, followed by three doses of cefazolin postoperatively. Outpatient antibiotics consisted of two days of amoxicillin-clavulanate and ciprofloxacin. Presenting vitals were within normal limits. Pertinent workup included a leukocytosis of 15.69 (Table 1) and CT abdomen pelvis demonstrating an ill-defined extraluminal air-fluid collection measuring 1.0 × 2.3 cm within the left pelvis likely originating from adjacent small bowel segment (Figure 1), markedly edematous urinary bladder wall with stranding of perivesical fat, filling defect involving left gonadal vein concerning for vessel thrombosis (Figure 2 and Figure 3), and marked mucosal edema involving multiple loops of the small and large bowel.

**Table 1:** Complete blood count (CBC) throughout hospitalization Hct, hematocrit; Hgb, hemoglobin; MCH, mean corpuscular hemoglobin; MCHC, mean corpuscular hemoglobin concentration; MCV, mean corpuscular volume; RBC, red blood cell; RDW, red cell distribution width; WBC, white blood cell

	Reference range and units	Day 1	Day 2	Day 3	Day 4	Day 5	Day 6	Day 7	Day 8	Day 9
WBC	3.8-10.8 thousand/uL	15.69(↑)	7.75	15.16(↑)	10.89	12.04(↑)	19.61(↑)	12.49(↑)	11.53(↑)	10.61
Hgb	11.7-15.5 g/dL	11.8(↓)	10.9(↓)	10.5(↓)	9.7(↓)	9.8(↓)	10.8(↓)	10.4(↓)	9.0(↓)	9.7(↓)
Hct	35.0-45.0 %	36.3	34.0(↓)	31.5(↓)	29.3(↓)	30.4(↓)	33.1(↓)	32.2(↓)	28.5(↓)	30.0(↓)
RBC	3.70-5.19 m/mcL	4.06	3.69(↓)	3.66(↓)	3.34(↓)	3.45(↓)	3.80	3.62(↓)	3.21(↓)	3.37(↓)
MCH	27.0-33.0 pg	29.1	29.5	28.7	29.0	28.4	28.4	28.7	28.0	28.8
MCHC	32.0-36.0 pg	32.5	32.1	33.3	33.1	32.2	32.6	32.3	31.6	32.3
MCV	80.0-100.0 fL	89.4	92.1	86.1	87.7	88.1	87.1	89.0	88.8	89.0
RDW	11.0-15.0 %	12.8	12.8	12.7	12.7	12.7	12.7	12.9	13.0	13.1
Platelets	140-400 thousand/uL	261	251	310	282	309	395	439	420	442

After obtaining blood cultures and urinalysis with urine culture, a seven-day course of broad-spectrum antibiotics consisting of vancomycin and piperacillin/tazobactam was initiated due to leukocytosis of unknown origin. A heparin infusion of therapeutic dosing for thrombi was concomitantly begun. Interventional radiology was consulted for possible percutaneous drainage of the fluid collection; however, this was deemed to be infeasible due to overlying structures. Despite the pharmacologic intervention, the patient began developing low-grade fevers in addition to worsening leukocytosis. Infectious disease was consulted in this setting, and the decision was made to obtain repeat imaging. CT of the abdomen and pelvis displayed small extraluminal air-fluid collection within the left pelvis in addition to trace volume extraluminal enteric contrast in the same region concerning contained perforation, presumed secondary inflammation involving the bladder and sigmoid colon, and left gonadal vein thrombus.

At this point, the patient consented to a diagnostic laparoscopy. In the operating room, a yellow-green discharge was noted to be coming from the vagina. After pneumoperitoneum was achieved, free fluid and edematous bowel adherent to the vaginal cuff and cul-de-sac were noted via direct visualization by laparoscope. The fluid was aspirated and sent for cultures. Blunt and hydrodissection were utilized in order to remove adhesions and mobilize the small bowel. These modalities are deemed to be safer than electrocauterization. General surgery and colorectal surgery had then arrived for an intraoperative consultation. After liberating the small bowel from the pelvis, general surgery was able to identify a small area with a serosal injury that was not amenable to primary repair. Prior to performing a small bowel resection, the bowel was run from the terminal ileum to the ligament of Treitz. With the exception of the known area of concern, the remaining bowel appeared unremarkable. With the assistance of colorectal surgery, a right proctoscopy was performed, which did not demonstrate any findings concerning a sigmoid colon or rectal injury. The portion of the bowel to be resected was then externalized from the abdomen. The area was once again assessed. In addition to the large serosal tear, a subcentimeter full-thickness injury on the antimesenteric border of the small bowel (not a result of the current surgery) was identified. Approximately 20 cm of non-salvageable ileum was resected, and the mesenteric defect was corrected.

The patient’s postoperative course was largely unremarkable. Vital signs stabilized, and the leukocytosis had resolved. The patient was discharged on postoperative day 4 with a two-day supply of amoxicillin-clavulanate and ciprofloxacin (for a total of seven-day antibiotic coverage) and 30 days of enoxaparin per attending preference. Blood and urine cultures obtained in the emergency department and fluid cultures obtained intraoperatively yielded negative results. 

## Discussion

The asymmetry of ovarian veins seems to play a significant role in the prevalence of OVT. The right ovarian vein typically drains directly into the IVC, whereas the left ovarian vein drains into the left renal vein [6]. This anatomical difference contributes to the predilection of OVT in the right ovarian vein, accounting for approximately 90% of cases [4]. The right ovarian veins' longer length and multiple incompetent valves, in conjunction with dextrorotation of the gravid uterus during pregnancy, further increase the risk of thrombosis [7]. These factors create conditions conducive to venous stasis and thrombus formation, therefore making the right ovarian vein more susceptible to thrombosis compared to the left ovarian vein.

OVT is often associated with hypercoagulable states, which increase the risk of thrombus formation. Conditions such as thrombophilias (e.g., factor V Leiden mutation), antiphospholipid syndrome, and malignancy can predispose individuals to OVT. Differential diagnoses include pelvic inflammatory disease, pyelonephritis, diverticulitis, and ovarian abscess; however, OVT was favored given the presenting history, recent surgery, and filling defect seen on imaging. Diagnosis of OVT typically involves imagining studies, with contrast-enhanced CT scans being the superior modality, offering nearly 100% sensitivity and specificity [8]. Treatment of OVT generally includes anticoagulation therapy to prevent further thrombus formation and antibiotics if infection is present [9,10]. In some circumstances, additional interventions such as thrombectomy or placement of an IVC filter may be warranted, especially if there is a risk of pulmonary embolism [5,9,10]. Early diagnosis and appropriate management are crucial to prevent complications and improve patient outcomes.

Despite its clinical significance, there are no specific guidelines or consensus on the optimal management of OVT. However, several studies suggest that treatment protocols for deep vein thrombosis (DVT) may be applicable. For instance, anticoagulation therapy, frequently used for DVT, has been recommended for OVT, especially when the thrombus is symptomatic or extends into adjacent veins. Studies have shown that a course of anticoagulation, typically lasting three months, can be effective in resolving OVT and preventing recurrence [4,10,11]. Data support the hypothesis that a history of OVT increases the risk of future OVT, with one study estimating recurrence to be three per 100 patient years [3,5]. There does not seem to be an official consensus on the utilization of imaging if a patient is asymptomatic. Additionally, the guidelines published by the American Society of Hematology pertaining to the management of VTE are often extrapolated to dictate OVT treatment due to the lack of specific OVT guidelines [7]. This approach underscores the need for further research in order to establish definitive treatment protocols for OVT.

This case report exemplifies a rare yet perilous complication of laparoscopic hysterectomy, namely septic pelvic thrombophlebitis (SPT). SPT is a condition that typically occurs as a result of pelvic vein endothelial damage, venous stasis, and hypercoagulability [8,11]. While it is most commonly associated with postpartum endometritis following cesarean deliveries in the setting of chorioamnionitis, it can also occur in other conditions, such as pelvic surgery or underlying malignancy [1,12]. In this specific case, the patient developed SPT following a total laparoscopic hysterectomy, bilateral salpingectomy, and a right ovarian cystectomy. Establishing the diagnosis posed difficulties due to the nonspecific symptomatology consisting of fever and abdominal pain, symptoms that are common manifestations of SPT. Despite the administration of antibiotics and anticoagulation, the patient failed to achieve clinical improvement, indicating the persistent nature of the condition [13]. The surgical technique, specifically the thermal damage induced by electrocautery, was proposed as a potential contributing factor to the development of OVT [6]. This highlights the importance of scrutinizing surgical techniques and their potential complications in the postoperative period. Although an incident report was not submitted, the physician made sure to express the utmost regret when explaining the outcome to the patient.

## Conclusions

The management of this patient required a multidisciplinary approach, with consultations from surgical specialists, infectious disease, and interventional radiology. Despite the challenges encountered while navigating this case, the patient's condition improved with continued antibiotics and anticoagulation with eventual return to her preoperative baseline. This case highlights the need for increased awareness regarding SPT as a potential complication of laparoscopic procedures. Surgeons should exercise extreme caution when working with electrocautery and utilize various instruments to isolate targeted structures in order to mitigate thermal damage. Preoperative VTE risk assessment calculators such as the Caprini score should also be employed to determine the need for anticoagulation. Given the relatively low incidence of reported SPT following laparoscopic gynecologic surgeries, further research is needed to provide more data on the risk of thromboembolism, which could ultimately improve treatment modalities and subsequent outcomes.
